# Chronic Sleep Restriction during Pregnancy - Repercussion on Cardiovascular and Renal Functioning of Male Offspring

**DOI:** 10.1371/journal.pone.0113075

**Published:** 2014-11-18

**Authors:** Ingrid L. B. Lima, Aline F. A. C. Rodrigues, Cássia T. Bergamaschi, Ruy R. Campos, Aparecida E. Hirata, Sergio Tufik, Beatriz D. P. Xylaras, Bruna Visniauskas, Jair R. Chagas, Guiomar N. Gomes

**Affiliations:** 1 Departamento de Fisiologia, Universidade Federal de São Paulo, São Paulo, SP, Brasil; 2 Departamento de Psicobiologia, Universidade Federal de São Paulo, São Paulo, SP, Brasil; 3 Departamento de Biociências, Universidade Federal de São Paulo, Santos, SP, Brasil; University of Southampton, United Kingdom

## Abstract

Changes in the maternal environment can induce fetal adaptations that result in the progression of chronic diseases in the offspring. The objective of the present study was to evaluate the effects of maternal chronic sleep restriction on blood pressure, renal function and cardiac baroreflex response on male offspring at adult age. Female 3-month-old Wistar rats were divided in two experimental groups: control (C) and chronic sleep restricted (CSR). Pregnancy was confirmed by vaginal smear. Chronic sleep restricted females were subjected to sleep restriction by the multiple platform technique for 20 h daily, between the 1^st^ and 20^th^ day of pregnancy. After birth, the litters were reduced to 6 rats per mother, and were designated as offspring from control (OC) and offspring from chronic sleep restricted (OCSR). Indirect blood pressure (BPi – tail cuff) was measured by plethysmography in male offspring at 3 months old. Following, the renal function and cardiac baroreflex response were analyzed. Values of BPi in OCSR were significantly higher compared to OC [OC: 127±2.6 (19); OCSR: 144±2.5 (17) mmHg]. The baroreflex sensitivity to the increase of blood pressure was reduced in OCSR [Slope: OC: −2.6±0.15 (9); OCRS: −1.6±0.13 (9)]. Hypothalamic activity of ACE2 was significantly reduced in OCSR compared to OC [OC: 97.4±15 (18); OSR: 60.2±3.6 (16) UAF/min/protein mg]. Renal function alteration was noticed by the increase in glomerular filtration rate (GFR) observed in OCSR [OC: 6.4±0.2 (10); OCSR: 7.4±0.3 (7)]. Chronic sleep restriction during pregnancy caused in the offspring hypertension, altered cardiac baroreflex response, reduced ACE-2 activity in the hypothalamus and renal alterations. Our data suggest that the reduction of sleeping time along the pregnancy is able to modify maternal homeostasis leading to functional alterations in offspring.

## Introduction

The shortening of sleeping time has become common in modern society. This alteration in sleep patterns appears to be attributable to extended working hours, longer and more frequent work shifts and the excessive use of computers and electronics [1]–[3]. Moreover, mechanisms that are essential for health are affected by sleep deprivation, resulting in changes such as reduced glucose tolerance [1], increased blood pressure, activation of the sympathetic nervous system [4], [5] and changes in hormonal pathways [6]–[8].

During pregnancy, sleep patterns change because of the physiological changes that are characteristic of this period [9]–[11]. These changes, together with increased workloads [12], [13] enhance the probability that pregnant women will experience sleep disturbances.

Studies have confirmed that changes such as nutritional restrictions [14], [15], diabetes [16], [17], or cortisol exposure [18], [19] during pregnancy are associated with changes in renal function and the development of hypertension in the adult offspring [20], [21]. Therefore, it is important to verify whether sleep pattern changes also influence fetal development. Permanent changes as a result of injuries during critical periods of fetal development are designated as “developmental programming of health and diseases” disease [22]. Although there are differences between human and murine nephrogenesis (i.e., in humans, it is completed by the 34th or 36th week of gestation [23], [24] whereas in rats, it continues during the early postnatal period [25]), the use of animal models is essential for advancing knowledge of human development.

In the last decade, sleep deprivation has been the subject of a number of studies; however, the impact of sleep disturbances during pregnancy on the subsequent offspring has only been evaluated in a small number of studies. Calegare et al showed that sleep deprivation in mice at the beginning of pregnancy resulted in important hormonal changes, fewer sustained pregnancies and altered expression of antioxidant enzymes in the offspring [8]. Thomal et al verified that sleep restriction in rats in the last week of pregnancy was associated with the development of hypertension and altered renal function in the offspring [26]. Nevertheless, no studies have evaluated the consequences of sleep restriction throughout the entire pregnancy (mimicking conditions of chronic sleep debt) for cardiovascular and renal functioning in the offspring.

The aim of the present study was to evaluate the parameters of renal and cardiovascular function with a focus on cardiac baroreflex sensitivity in offspring from rats that were subjected to sleep restriction throughout their pregnancies.

## Materials and Methods

The present study was approved by the Ethical Research Committee of the Universidade Federal de São Paulo - UNIFESP (Permit number: 1170/10) and followed international guidelines for the care of research animals.

### Obtaining offspring

To obtain the litters, 3-month-old female and male Wistar rats from our colony, weighing 200–250 g and 300–350 g, respectively, were used. The females in proestrus were caged overnight with a male, and vaginal smears were taken the following morning. A positive smear was considered day 0 of gestation. The pregnant females were divided into two groups: C (the control mothers, n = 8) and CSR (the chronically sleep restricted mothers, n = 8). Both groups had free access to water and food for the full experimental period. The animals were maintained in a room with controlled temperature (21±2°C, 60%) on a 12:12 h light/dark cycle with lights on at 07:00 hours. After pregnancy was confirmed, the females were transferred to a sleep restriction room under the same conditions. During pregnancy, the body weights of the C and the CSR groups were evaluated once a week.

### Sleep restriction

The sleep restriction technique was based on the muscle atony that accompanies paradoxical sleep [27]. Briefly, 10 narrow circular platforms (6.5 cm in diameter) were placed inside a tiled tank (123×44×44 cm) filled with water to within 1 cm below the upper border of the platform. In the CSR group, 2 to 6 rats were placed on the platforms in an arrangement that allowed them to move inside the tank and jump from one platform to the other. Two days before the beginning of the study, the animals were adapted to the water tank for a period of 1 h to avoid unnecessary drops in the water. On the day pregnancy was confirmed, the CSR animals were placed in the tank at 14:00 h, and the next day at 10:00 h, they were placed back in their home cages, where they could sleep freely and had free access to food and water. This procedure was repeated until the 20^th^ day of pregnancy. At 10:00 h on the 20^th^ day of pregnancy, the CSR rats were placed back in their home cages and were maintained there to await spontaneous parturition and weaning of the offspring. The animals in the C group remained in their home cages in the same room where the sleep deprivation took place.

### Experimental groups

After the births, the litters were reduced to 6 offspring (preferably 4 male and 2 female) that stayed with the mothers for 28 days. This procedure was intended to avoid differences during lactation. Only male offspring were studied in the present study (the female offspring were used for another study). The size of the litters mainly varied between 10 and 12 offspring. After weaning, the rats were placed in collective cages and were divided into two groups: the OC, the offspring from the control mothers, and the OCSR, the offspring from the chronically sleep restricted mothers. The groups had water and food *ad libitum*. OC and OCSR body weights were evaluated from birth until three months of age. The offspring (OC and OCSR) underwent different experimental procedures: 1) evaluation of renal function, 2) evaluation of cardiovascular function and baroreflex response, and 3) analysis of the angiotensin converting enzyme (ACE) and angiotensin converting enzyme 2 (ACE2) activity in the hypothalamus and kidneys. All rats within each experimental procedure were from different mothers.

### Measurement of arterial blood pressure – indirect method

The arterial blood pressure of the offspring was evaluated by tail plethysmography (IITC Life Science Inc.; Woodland Hills, CA, USA) at 3 months of age. Two weeks before the measurements were taken, the rats were conditioned to the procedure by being placed in the restrainer tube of a chamber that was kept at 33–34°C, followed by inflating the cuff after placing it at the base of the tail.

### Renal function evaluation

Renal function was studied in the 3-month-old offspring (10 rats in the OC group and 7 rats in the OCSR group). The animals were anaesthetized with sodium thiopental (30 mg/Kg) and placed on a warmed table to maintain body temperature at 37°C. Tracheotomy was performed, followed by insertion of polyethylene catheters into the jugular vein for infusions and into the carotid artery for blood sampling. Urine was collected through a catheter inserted into the bladder. Thirty minutes after the surgical procedures, the animals received 1 ml of saline containing inulin (300 mg/kg) and sodium para-aminohippurate (PAH, 6.66 mg/kg), and then an infusion began of 0.1 ml/min of saline containing inulin (5 mg/min/kg) and PAH (1.33 mg/min/kg). Thirty minutes later, the first urine and blood collection was taken (2 or 3 collections were performed in each experiment). Plasma and urine inulin and PAH concentrations were measured by colorimetry in order to estimate the glomerular filtration rate (GFR) [28] and renal plasma flow (RPF) [29]. The Na+ and K+ concentrations in the plasma and urine were evaluated using the Buck Scientific Model 910 Flame Photometer (São Paulo, Brazil).

Renal vascular resistance (RVR) was calculated from the pressure-to-flow ratios using the equation RVR  =  mean blood pressure before renal function study/renal blood flow (RBF), as previously described [30]. RBF was calculated using the equation RBF  =  RPF/(1 - hematocrit). To determine hematocrit levels, samples of arterial blood (100 µl) were collected in glass capillary tubes (Tecnic Glass for micro-hematocrit) and were centrifuged for 15 min at 4000 rpm. After centrifugation, the percentages of red blood cells were calculated [30].

### Cardiovascular parameters and analysis of baroreceptor reflex sensitivity in conscious rats

Cardiovascular function was studied in 3-month-old offspring (9 rats in the OC group and 9 rats in the OCSR group). For the intravenous injection of drugs and direct recording of the arterial blood pressure, the rats were anesthetized with ketamine and xylazine (40 mg/kg and 20 mg/kg, i.p., respectively) (Vetbrands, Jacareí, Brazil) through catheters inserted in the femoral vein and artery. After 24 h of surgical recovery, cardiovascular parameters, systolic arterial pressure (SAP); diastolic arterial pressure (DAP); mean arterial pressure (MAP); and heart rate (HR) signals, were recorded in freely moving rats using an analog-digital board with PowerLab software (AD Instruments, Australia).

Subsequent to the measurements of arterial pressure and heart rate, pressor doses of phenylephrine (3, 5 and 10 µg/kg, IV -Sigma-Aldrich) and depressor doses of sodium nitroprusside (5, 15 and 20 mg IV Sigma-Aldrich) were randomly acutely administered at 15-minute intervals to induce progressive increases or decreases in blood pressure and bradycardia or tachycardia reflexes. The sensitivity of the arterial baroreceptor for HR control was evaluated by the mean index that related changes in HR to changes in MAP and was expressed as beats per mmHg (bpm/mmHg).

### Angiotensin converting enzyme (ACE) and angiotensin converting enzyme 2 (ACE2) activities in the hypothalamus and kidneys

To extract the protein, tissues were homogenized in a 50 mM Tris-HCl buffer, pH 7.4, containing NaCl 100 mM and 0.1% Triton. Homogenates were centrifuged at 1,000 *g* for 15 min at 4°C, and the supernatant was frozen at −20°C until the ACE activity measurements. The protein content was measured with Bradford's [31] method using bovine serum albumin as the standard.

ACE and ACE2 activities in the tissue extracts was determined using, respectively, the FRET peptides Abz-FRK(Dnp)P-OH and MCA-APK(Dnp)-OH (Aminotech Pesquisa e Desenvolvimento, Brazil). ACE activity assays were performed in a Tris-HCl 100 mM pH 7.0 buffer containing NaCl 100 mM and ZnCl_2_ 10 µM [32] and ACE2 activity in 75 mM Tris-HCl pH 6.5 buffer, containing NaCl 1 M, ZnCl_2_ 0.5 mM [33]. Lisinopril (Sigma, USA) and DX600 (Proteimax, Brazil) were used as ACE and ACE2 inhibitors, respectively, to ensure substrate specificity. The reactions were continuously followed in a Gemini XS fluorimeter (Molecular Devices Company, Sunnyvale, CA, USA) that measured the fluorescence at λ_ex_ = 320 nm and λ_em_ = 420 nm (Abz group) and λ_ex_ = 360 nm and λ_em_ = 440 nm (MCA group). Measurements were performed in duplicate and ACE activity values were reported as nanomoles of substrate hydrolyzed per minute per milligram of protein (nM.min^−1^.mg^−1^).

### Statistical analysis

All values are expressed as the mean ± SEM. Prisma (Graph Pad Software) was used for statistical analysis. Data were analyzed using the unpaired Student's T-test.

## Results

**Figure 1:**
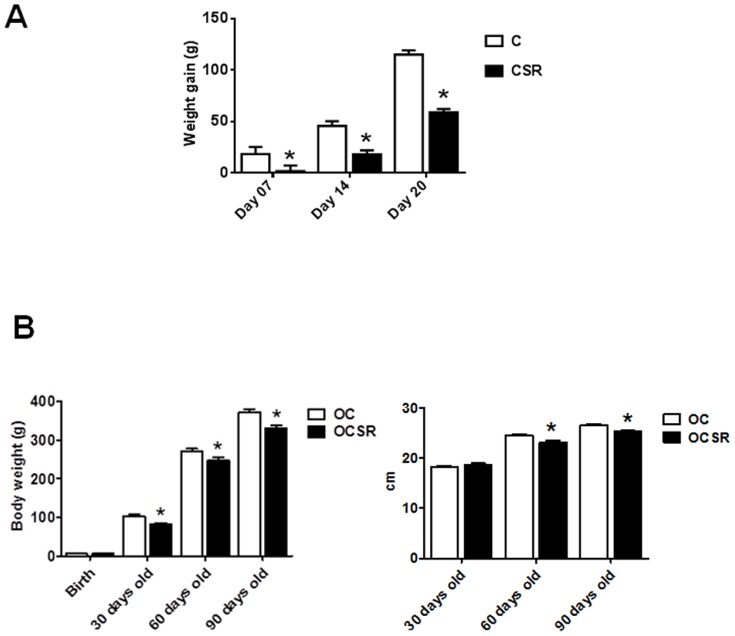
Weight gain during pregnancy, C refers to the control mothers and CSR to the chronically sleep restricted mothers. (A). Body weights (left) and naso-anal measurements (right) of the OC (offspring from the control mothers) and from the OCSR (offspring from the chronically sleep restricted mothers) groups (B). Data are reported as the means ± SEM. * p≤0,05 vs. the C group (Student's t-test).

Figure 1A shows the pregnancy weight gains of the C and the CSR rats. The chronically sleep-restricted rats gained less weight after the first week of pregnancy compared with the C group. Figure 1B shows the body weights of the offspring from birth until 3 months old. We did not observe differences in body weight at birth (OC: 5.6±0.08 g (18); OCSR: 5.9±0.17 g (26). However, beginning at 1 month, the OCSR body weights were lower in comparison with those of the OC group, a trend that continued until age 3 months.

**Figure 2:**
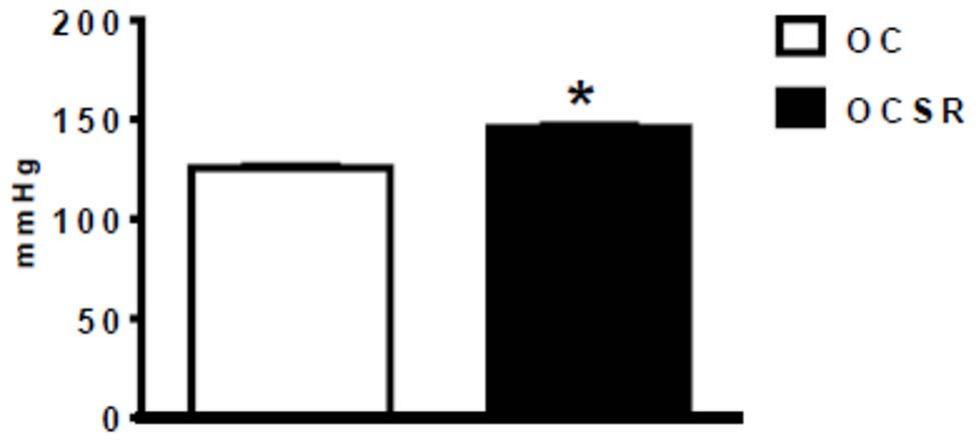
Systolic blood pressure – indirect measurements. OC [the offspring from the control mothers, 127±2.6 (19)]; OCSR [the offspring from the chronically sleep restricted mothers, 144±2.6 (17)] * p≤0.05 vs. the OC group (Student's *t*-test).

The arterial blood pressure (BPi) values measured in the 3-month-old rats using the indirect plethysmographic method were significantly increased in the OCSR group in comparison with the OC group (Figure 2 – OC: 127±2.63 mmHg; OCRS: 144±2.59 mmHg).

Glomerular filtration rate (GFR) was increased in the OCSR group compared with the OC group (Table 1). Sodium and potassium plasma concentrations were similar in both groups. Moreover, there were no significant differences in the fractional excretions of sodium and potassium in the studied groups (Table 2).

**Table 1:** Renal function parameters in the studied groups.

	V (ml/min/kg)	GFR (ml/min/kg)	RPF (ml/min/kg)	RVR (mmHg/ml.min.kg)
**OC** (10)	0.123±0.01	6.42±0.2	21.46±1.1	2.72±0.15
**OCSR** (07)	0.136±0.01	7.41±0.3*	23.41±2.7	3.38±0.6

Data are reported as the means ± SEM. The number of animals is indicated in parentheses. OC refers to the offspring from the control mothers; OCSR refers to the offspring from the chronically sleep restricted mothers; V, urinary flow; GFR, glomerular filtration rate; RPF, renal plasma flow.

*p≤0.05 vs. the C group (Student's *t*-test).

**Table 2:** Evaluation of sodium and potassium excretion.

	P_Na_ (mEq/l)	FE_Na_ (%)	EA_Na_ (µEq/min/kg)	P_K_ (mEq/l)	FE_K_ (%)	EA_K_ (µEq/min/kg)
**OC** (10)	140±0.9	1.2±0.1	10.8±1.0	3.2±0.12	35.8±1.5	7.6±0.5
**OCSR** (07)	140±0.7	1.3±0.3	14.1±3.1	3.1±0.04	37.7±7.1	8.9±1.8

Data are reported as the means ± SEM. The number of animals is indicated in parentheses. OC refers to the offspring from the control mothers; OCSR, to the offspring from the chronically sleep restricted mothers; PNa, plasma sodium concentration; FENa, fractional excretion of sodium; EANa, excreted amount of sodium; P_K_, plasma potassium concentration; FE_K_, fractional excretion of potassium; EA_K_, excreted amount of potassium.

*p≤0.05 vs. the C group (Student's *t*-test).

**Figure 3:**
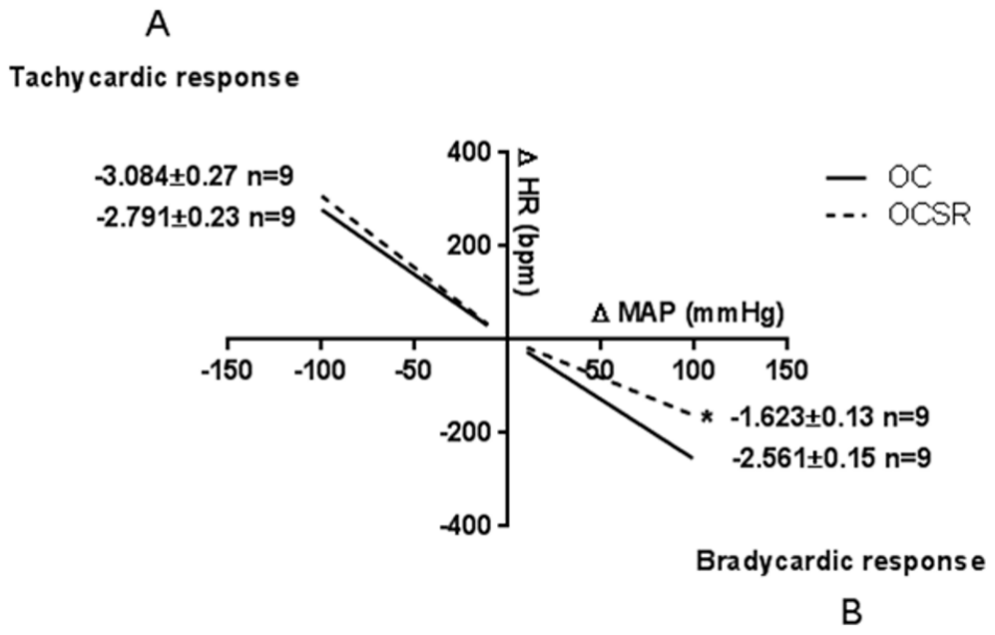
Baroreceptor reflex sensitivity. (**A**): Reflex heart rate response (ΔHR) to the decreased mean arterial pressure (ΔMAP) induced by sodium nitroprusside (180 µg/ml, i.v.). (**B**): Reflex heart rate response (ΔHR) to the increased mean arterial pressure (ΔMAP) induced by phenylephrine (100 µg/ml, i.v.). Data are reported as the means, and the number of animals is indicated in parentheses. OC refers to the offspring from the control mothers; OCSR, refers to the offspring from the chronically sleep restricted mothers. *P<0.05 versus OC (Student's t-test).

Resting heart rates and systolic, diastolic and mean arterial blood pressures are shown in Table 3. The baroreceptor reflex sensitivity data that were obtained are shown in Figure 3. The OCSR group presented an impaired response to phenylephrine (Fig 3B) but not to sodium nitroprusside infusion (Fig 3A).

**Table 3:** Resting heart rates (HR) and systolic (SAP), mean (MAP) and diastolic (DAP) arterial pressures (direct measurements).

	HR (bpm)	SAP (mmHg)	MAP (mmHg)	DAP (mmHg)
**OC** (9)	362±12	121±2.5	99±3.1	89±3.4
**OCSR** (9)	380±7	138±3.3*	111±2.7*	98±3.2

Data are reported as the means ± SEM. The number of animals is indicated in parentheses. OC refers to the offspring from the control mothers; OCSR refers to the offspring from the chronically sleep restricted mothers.

The data on ACE and ACE2 activities are shown in Table 4. The hypothalamic ACE2 activity was significantly reduced in the OCSR group compared with the OC rats. However, no significant changes were observed in the hypothalamic ACE activity or in the renal ACE and ACE2 activity.

**Table 4:** ACE and ACE2 activity in hypothalamus and kidney extracts.

	ACE	ACE	ACE2	ACE2
	kidney nM/min/mg	hypothalamus nM/min/mg	kidney nM/min/mg	hypothalamus nM/min/mg
**OC**	0.41±0.1 (12)	1.16±0.2 (18)	24.9±3 (12)	97.4±15 (18)
**OCSR**	0.47±0.1 (9)	1.14±0.06 (17)	19.5±2 (11)	60.2±3,6 (16) *

## Discussion

The present study showed that chronic sleep restriction during pregnancy caused hypertension, reduced sensitivity of the baroreflex response and changes in renal function in the resulting offspring. The development of hypertension may be related to disturbances in the central regulation of blood pressure. Nevertheless, variations in the baroreflex control of the heart could be either a cause or a consequence of the hypertension. Although renal filtration in the experimental group changed, we did not observe sodium retention, at least at the time the study was performed. Thus, the development of hypertension in this experimental model appears to be unrelated to inappropriate sodium handling.

In a recent study from our laboratory, we evaluated the repercussion of sleep restriction on the last week of pregnancy in rats, on the offspring [26]. In the present study, sleep restriction began as soon as pregnancy was confirmed in order to better mimic real-world, modern-day chronic sleep deprivation conditions. In both studies, the experimental group showed less weight gain during pregnancy, most likely owing to the stress caused by the sleep restriction [34]. Sleep restriction or deprivation is related to nonspecific stress, which may interact with the overall effects of sleep loss *per se* [26]. The lower weight gain during pregnancy did not lead to reduced birth weights in the offspring.

We observed in the present study that chronic sleep restriction during pregnancy also resulted in the development of hypertension in the offspring. This change was observed in the offspring of dams that were subjected to sleep restriction during the last week of pregnancy [26] suggesting that either chronic sleep restriction (throughout the entire pregnancy) or restriction only at the end of pregnancy leads to changes in the fetus that result in the altered regulation of blood pressure in the adult offspring. To better understand this change, we studied the parameters of 1) renal function, including sodium excretion and 2) cardiovascular function, focusing on cardiac baroreceptor sensitivity.

Renal function was evaluated to verify whether hypertension was associated with a decreased ability to eliminate sodium and volume loads. The results showed that in the experimental conditions of this study, the OCSR was able to excrete sodium loads at the same magnitude as that of the OC group, suggesting that this mechanism is not the primary cause of the hypertension. However, increased glomerular filtration rates (GFR) were observed in the OCSR. Under normal conditions, a sudden increase in blood pressure is not transmitted to the glomerular capillaries because of the kidneys' autoregulatory mechanisms, which prevent GFR changes. However, during hypertension, the autoregulation mechanism may be impaired, and pressure in the glomerular capillaries may increase [35]. It is possible that autoregulation was altered in the OCSR group, elevating the blood pressure in the glomerular capillaries and increasing the GFR [35]. In addition, in the OCSR there was a slight increase in renal vascular resistance. Afferent and efferent arterioles are important components of GFR regulation. The resistance of these arterioles is affected by a number of vasoactive compounds including angiotensin II (AngII) [36]. Angiotensin II is known to affect both afferent and efferent resistance, but it appears to predominantly affect the efferent arterioles, thereby increasing glomerular hydraulic pressure and filtration fraction [37], [38]. ACE and ACE 2 activity were measured in an attempt to confirm the participation of the renin-angiotensin system in the changes in glomerular function, but we did not observe significant differences in either ACE or ACE2 activity. In the kidneys, vasoactive substances (such as nitric oxide, dopamine and prostaglandins) counteract the effect of AngII to avoid excessive vasoconstriction. It is possible that changes in these substances could contribute to elevating the GFR, but additional experiments are necessary to clarify this hypothesis.

The baroreflex control of the heart rate was evaluated by analyzing the responses to sudden changes in blood pressure after the administration of *phenylephrine* or sodium nitroprusside. Altered baroreflex sensitivity was observed in the OCSR group in comparison with the OC group, suggesting the deregulation of this mechanism.

Dysfunction in baroreflex control has been observed in complex diseases such as hypertension and diabetes [39], [40]. However, the role of baroreceptors in chronic cardiovascular diseases is still a matter of discussion and study. Salgado and Krieger (1973) showed that complete adaptation of the baroreceptors occurred after roughly two days of maintained hypertension. In addition to the baroreceptors adaptation to the increased blood pressure, in hypertension, there is also reduced baroreflex sensitivity, which impairs the reflex regulation of blood pressure [41]. However, in the present study, it was not possible to establish whether the baroreceptor impairment was a cause or a consequence of the hypertension.

In general, reduced vascular elasticity and structural changes in large arteries are identified as key mechanisms in the adaptation and impairment of baroreceptor reflex control in hypertension. However, ionic and paracrine factors that interfere with baroreceptor activity (peripheral sensory mechanisms) and with the central mediation of the baroreflex response have also been shown to contribute to the impaired response [42]. Sleep deprivation and restriction can alter the regulation of a number of hormones [43], [44]. Schussler et al. showed that sleep deprivation results in the increased secretion of ghrelin, ACTH, cortisol and GH. Recently, it was demonstrated that sleep deprivation can increase the production of hypothalamic hormones such as prepro-orexin (PPO) and neuropeptide Y (NPY), in addition to increasing the plasma concentration of glucagon, corticosterone and norepinephrine [45]. Calegare et al. studied the influence of sleep deprivation at the beginning of pregnancy on hormonal profiles and observed significantly reduced progesterone plasma concentrations and increased corticosterone concentrations in the deprived groups compared with the controls. Hormonal disarrangements during critical ontogenetic periods may act as “endogenous functional teratogens”, which may be responsible for the appearance of hypothalamic dysfunction in offspring [46], [47]. In view of the fact that hormonal changes during pregnancy can be related to central imbalances that can lead to hypertension, we measured ACE and ACE2 activity, components of the renin-angiotensin system (RAS) in the total tissue extracts from the hypothalami of the studied groups. All components of the renin-angiotensin system (RAS) are present in the central nervous system (CNS). Angiotensin II, in addition to its vasoconstrictor effect, is known to increase the production of mitochondrial ROS. An excess of this substance in the brain contributes to increased sympathetic outflow and reduces baroreflex sensitivity [48]. In another way, the Ang-(1–7) may counteract the effects of ROS contributing to maintain normal blood pressure. The balance between AngII and Ang-(1–7) within the CNS contributes to maintaining normal blood pressure and the proper functioning of the arterial baroreceptor reflex in controlling the heart rate [48]. Systemic AngII can also access the CNS through the circumventricular organ, where the blood-brain barrier is more permeable, to reach areas of the brain stem and hypothalamus such as the paraventricular nucleus (PVN), contributing to sympathoexcitation and hypertensive response [49]–[51]. The expression of RAS components in the PVN was studied by Sriramula et al (2013) in AngII-infused hypertensive rats. In these animals, the authors observed reduced mRNA and reduced ACE2 protein expression in the PVN. Moreover, in hypertensive mice with elevated levels of AngII, the reduced central ACE2 activity was associated with impaired baroreflex sensitivity [52]. In this experimental model, ACE2 overexpression in the CNS restored baroreflex sensitivity and reduced hypertension, confirming the role of central ACE2 in preserving baroreflex function.

In our experimental model, hypothalamic ACE2 activity was significantly reduced in the hypertensive offspring (the OCSR group), suggesting that a central imbalance in the RAS components could have been involved in the changes observed in this study.

Thus, it is possible that changes in the maternal environment during fetal development are associated with central modifications that persist to some degree in adulthood to generate cardiovascular and renal disorders in the offspring. However, more studies are needed to identify possible mediators of the changes found in this study.
